# Maintained complete response to talazoparib in a *BRCA-2* mutated metastatic luminal breast cancer: case report and review of literature

**DOI:** 10.3389/fonc.2023.1158981

**Published:** 2023-05-05

**Authors:** Víctor Albarrán, Jesús Chamorro, Javier Pozas, María San Román, Diana Isabel Rosero, Cristina Saavedra, María Gion, Alfonso Cortés, Elena Escalera, Eva Guerra, Elena López Miranda, María Fernández Abad, Noelia Martínez Jañez

**Affiliations:** Medical Oncology Department, Ramon y Cajal University Hospital, Madrid, Spain

**Keywords:** breast cancer, *BRCA*, germline, PARP inhibitors, olaparib, talazoparib

## Abstract

PARP inhibitors are progressively becoming a part of our therapeutic arsenal against BRCA-defective tumors, because of their capacity to induce synthetic lethality in cells with a deficiency in the homologous recombination repair system. Olaparib and talazoparib have been approved for metastatic breast cancer in carriers of germline BRCA mutations, which are found in approximately 6% of patients with breast cancer. We report the case of a patient with metastatic breast cancer, carrier of a germline mutation in BRCA2, with a complete response to first-line treatment with talazoparib, maintained after 6 years. To the best of our knowledge, this is the longest response reported with a PARP inhibitor in a BRCA-mutated tumor. We have made a review of literature, regarding the rationale for PARP inhibitors in carriers of BRCA mutations and their clinical relevance in the management of advanced breast cancer, as well as their emerging role in early stage disease, alone and in combination with other systemic therapies.

## 1 Introduction

Breast cancer (BC) accounts for approximately 30% of malignancies in women worldwide (1), with an incidence ranging from 27 in 100000 (Africa and East Asia) to 97 in 100000 (North America and Western Europe), reflecting its association with lifestyle and social factors (2). Metastatic BC remains a virtually incurable disease and is still the leading cause of cancer-related death in women globally (3), though the prognosis of this condition has been improved by the incorporation of novel therapies beyond conventional chemotherapy (CT).

Around 10% of malignant breast tumors are associated with a genetic predisposition (3). Although several breast cancer susceptibility genes have been identified, the most common germline mutations that lead to a family history of BC affect *BRCA1* and *BRCA2*. Pathogenic variants in these genes are associated with an increased risk of several tumors, being the strongest hereditary risk factors for breast and ovarian cancers. A contemporary prospective cohort study with 9856 carriers of pathogenic *BRCA* variants, reported a cumulative BC risk to 80 years of 72% in *BRCA1* mutation carriers (95% confidence interval [CI] 65-79%) and 69% in *BRCA2* mutation carriers (95% CI 61-77%) (4). Germline *BRCA2* mutation is more present in the ER+/HER2- population compared to *BRCA1* (5).

The biological functions of *BRCA1* and *BRCA2* are related to the repair of DNA double-strand breaks (DSBs) by homologous recombination (HR), whereas PARP is an enzyme involved in base excision repair, which is key to repair of DNA single-strand breaks (SSBs). The blockade of PARP function causes an increase in SSBs, which are converted during cell replication to DSBs -usually repaired by HR-. In *BRCA1/2* defective cells, the inhibition of PARP leads to the accumulation of DSBs that cannot be repaired due to a deficiency in the HR system, causing cell death, a phenomenon known as synthetic lethality. This is the biological rationale for the use of PARP inhibitors (PARPi) in *BRCA*-defective tumors.

*BRCA1/2* mutations are classified as ESCAT I/OncoKb I actionable alterations. PARPi have been approved by the FDA for the treatment of four BRCA-associated tumors. In ovarian cancer, several drugs (olaparib, niraparib, rucaparib) have demonstrated clinical benefit both in recurrent tumors and as maintenance treatment after first-line CT in platinum-sensitive disease (6–12). In pancreatic and castrate-resistant prostate cancers, olaparib has been approved for patients with mutations in *BRCA* and other HR-related genes (13, 14).

In breast cancer, both olaparib and talazoparib have received FDA approval for carriers of *BRCA* mutations (pathogenic or likely pathogenic variants) with metastatic Her2-negative disease, based on the results from the OlympiAD (15) and EMBRACA (16) trials, respectively. In the phase III trial BROCADE-3 (17), addition of PARPi (veliparib) to an active platinum doublet (carboplatin/paclitaxel) resulted in significant improvement in progression-free survival (PFS) in patients with metastatic BC and germline *BRCA* mutations.

## 2 Case report

Our patient was a 38-year-old female, with no relevant previous medical history -except for mild bronchial asthma and chronic treatment with low-dose steroids, due to primary adrenal insufficiency-. Regarding family history, her mother had been diagnosed with breast cancer at 42 years of age, her father had been diagnosed with prostate cancer at 78 years, and her maternal aunt had been diagnosed with breast and ovarian cancer at 46 years.

In September 2014, the patient consulted for a palpable lesion, approximately 3 cm in size, in the inner upper quadrant of her right breast. Physical examination confirmed the lesion and did not reveal skin retraction, ulceration, tangible lymph nodes, or any other pathological findings. Mammogram, breast echography and breast magnetic resonance imaging (MRI) revealed the presence of a highly suspicious mass with irregular margins, with a maximum diameter of 32 mm, together with two satellite nodules approximately 8 mm in size. No pathological lymph nodes were identified.

A core needle biopsy was performed, which confirmed the diagnosis of multifocal infiltrating ductal carcinoma, with histologic grade 3 and a Ki67 proliferation index of 90%. Estrogen and progesterone receptor expression was detected in 70% and 15% of the cells, respectively. Immunohistochemical analysis revealed intense expression of E-cadherin and cytokeratin 19, with no Her2 overexpression (score 0+). Considering her family history and her age at diagnosis, the patient was referred to the hereditary cancer unit. She and her mother underwent a genetic study that revealed a pathogenic germline mutation in *BRCA2*.

In October 2014, she started neoadjuvant treatment with 5-fluorouracil, epirubicin and cyclophosphamide for 4 cycles (FEC scheme), followed by 8 cycles of weekly paclitaxel. In April 2015, she underwent bilateral mastectomy, with no relevant surgical complications. Histological examination of the surgical piece demonstrated pathological complete response [grade 5 of the Miller and Payne system (18)]. She started adjuvant hormone therapy with tamoxifen in May 2015 and began usual post-treatment surveillance. In January 2016, the patient underwent prophylactic bilateral oophorectomy after detailed genetic counseling.

In October 2016, she consulted with a rapidly growing lump on the right side of her head. A core needle biopsy was performed, demonstrating bone relapse of breast carcinoma, with positive expression of estrogen and progesterone receptors. The score for the immunohistochemical determination of Her2 was 2+, but gene amplification was discarded by fluorescence *in situ* hybridization (FISH).

**Figure 1:**
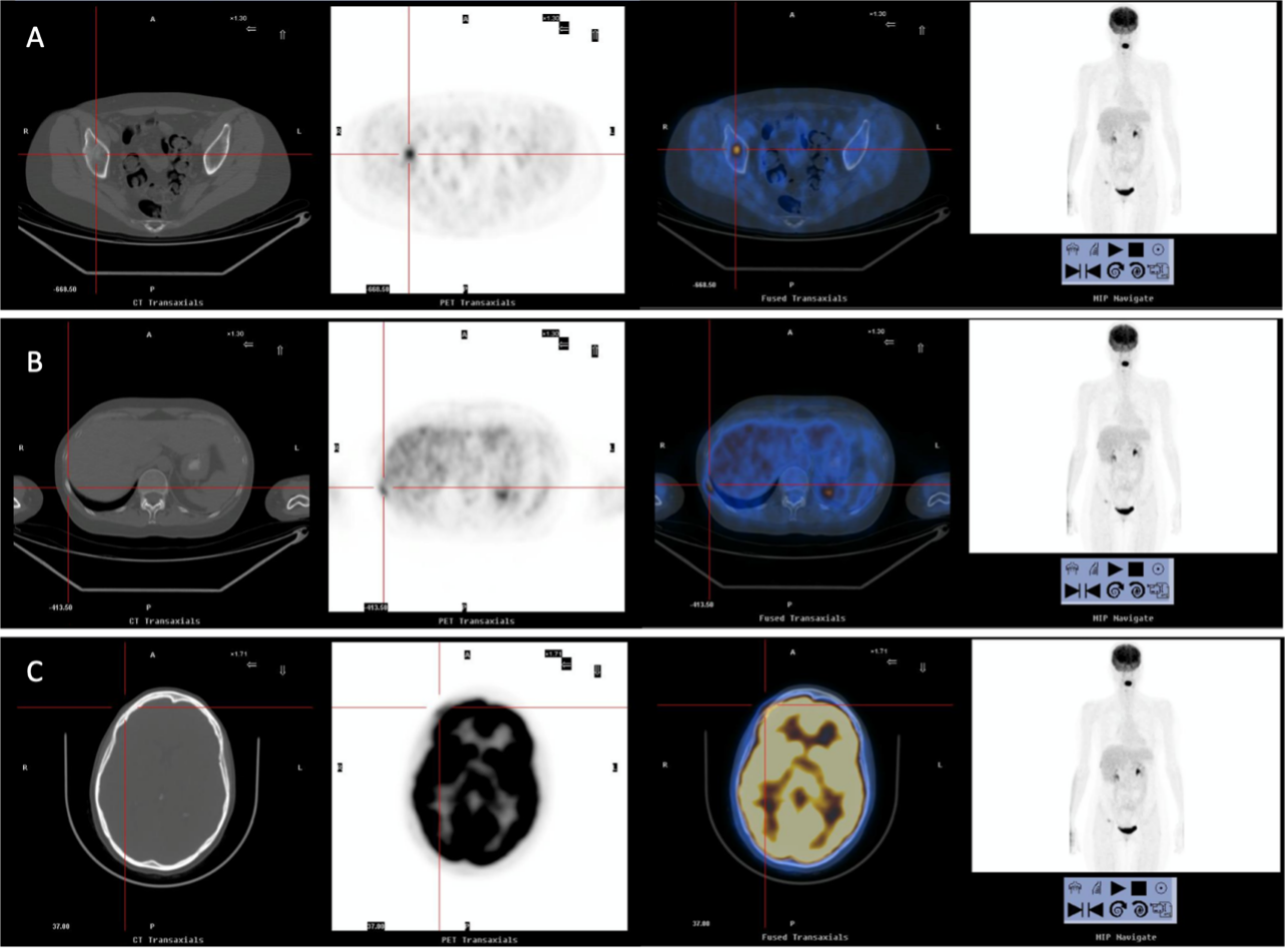
Images from computerized tomography (CT), FDG-PET and bone scintigraphy, showing bone relapse in right acetabulum **(A)**, tenth right rib **(B)** and right frontal bone **(C)**.

Whole-body positron emission tomography with 18F-fluorodeoxyglucose (FDG-PET) revealed pathological deposits of radiotracer, with high metabolic activity, in the right frontal bone, corresponding with the biopsied lesion, the right acetabulum and the tenth right rib, all of which were suggestive of tumor viability (**Figure 1**), as well as two non-specific liver nodular lesions. Bone scintigraphy confirmed the bone metastases, and MRI confirmed the metastatic nature of the liver nodules. These findings led to a diagnosis of stage IV hormone-receptor (HR)+ breast cancer with bone and liver infiltration.

Due to the early relapse of the disease while on adjuvant treatment with tamoxifen, the patient declined hormone therapy, alone or in combination with a cyclin-dependent kinase inhibitor (CDKi). After being informed about the non-curative intent and potential adverse effects of conventional CT, the patient discarded this option, and asked about additional therapeutic options. Preliminary data regarding the promising results of PARP inhibitors in untreated BRCA mutation carriers with BC (19–21), were discussed with the patient, and talazoparib was solicited as a compassionate drug.

**Figure 2:**
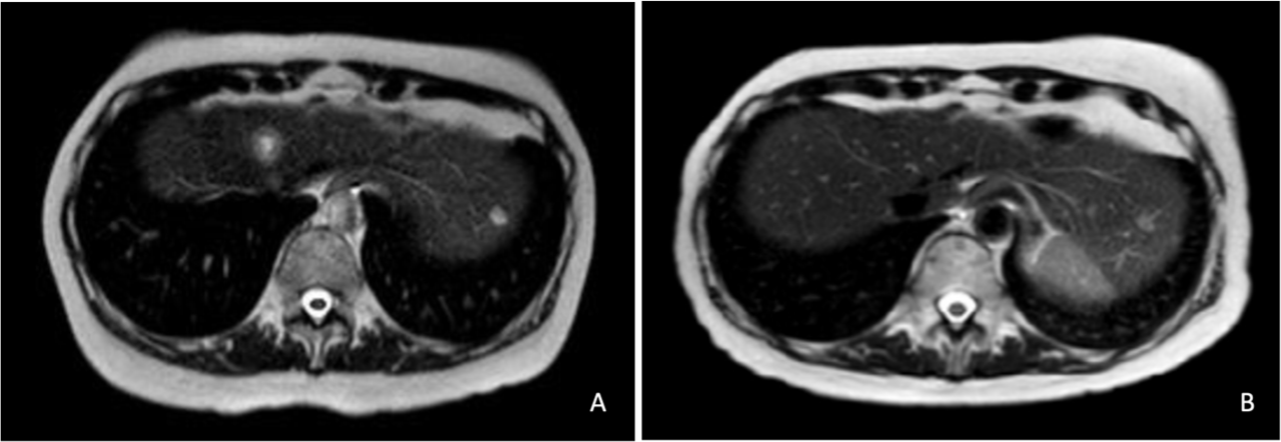
MRI study showing two liver metastases in January 2017 **(A)**. Complete response of liver lesions after 2 cycles of talazoparib in April 2017 **(B)**.

**Figure 3:**
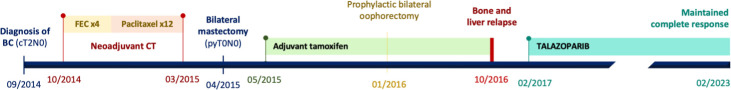
Timeline of the case.

She started treatment with talazoparib 1 mg/24 h in February 2017, achieving a complete clinical and metabolic response after two cycles. In April 2017, bone scintigraphy and liver MRI showed no evidence of disease (**Figure 2**). She has completed 90 cycles of treatment to date, with good tolerance, except for mild hematologic toxicity (grade 1 anemia). The disease remains in complete response on the last FDG-PET scan, performed in November 2022. A timeline of the case is presented in **Figure 3**.

## 3 Discussion

### 3.1 PARPi in metastatic BC

The OlympiAD (15) was a phase 3 clinical trial that compared olaparib (300 mg twice daily) with standard non-platinum single-agent CT (eribulin, capecitabine or vinorelbine) in 205 patients who were carriers of g*BRCA*m with metastatic Her2-negative BC (randomization 2 to 1). Among the patients in the experimental group, 57.1% had mutations in *BRCA1*, 41.0% had mutations in *BRCA2* and 2.0% had mutations in *BRCA1* and *BRCA2* simultaneously. The patients had received no more than two previous lines of CT (12.7% had new metastatic BC and 71.2% had been previously treated with CT in the olaparib group). In the olaparib group, 77.6% of patients had two or more metastatic sites. Among the patients who received olaparib, 50.2% had HR+ tumors and 41.0% had triple-negative tumors. The median PFS, set as primary endpoint, was significantly longer in the olaparib group (7.0 months vs 4.2 months; HR 0.58, *p <*0.001). Patients in the olaparib arm achieved a median overall survival (OS) of 19.3 months, versus 17.1 months in the control arm (*p* = 0.51). The objective response rate (ORR) was 59.9% in the olaparib group (28.8% in the control group). The experimental group also appeared to have a favorable rate of grade 3-4 adverse events and treatment discontinuation. An analysis of OlympiAD patients treated in the first-line setting demonstrated a survival benefit with olaparib, suggesting a higher benefit with the earlier use of PARPi (22).

In the phase 3 trial EMBRACA (16), 431 patients with advanced BC and g*BRCA*m were randomly assigned (2:1) to receive talazoparib (1 mg once daily) or standard single-agent CT (eribulin, capecitabine, vinorelbine, or gemcitabine). Of the patients treated with talazoparib, 42.9% harbored mutations in *BRCA1*, and 51.2% of them had mutations in *BRCA2*. The patients had received no more than three previous lines of CT (38.7% had new metastatic BC, 37.3% had been treated with 1 prior regime of CT, and 24% had received 2 or 3 previous lines of CT). In the talazoparib group, 45.3% were triple-negative and 54.7% HR+ tumors based on the most recent biopsy. The experimental group obtained a higher median PFS (8.6 months vs 5.6 months; HR 0.54; *p* < 0.001), a higher median OS (22.3 months vs 19.5 months; HR 0.76; *p* = 0.11) and a higher ORR (62.6% vs 27.2%) -complete response in 5.5% and partial response in 57.1% of patients-. Hematologic grade 3-4 adverse events were more frequent with talazoparib (55% vs. 38%), although globally tolerance, quality-of-life reports, and time to clinical deterioration favored the experimental group.

The use of a PARP inhibitor in the first-line setting, due to the unwillingness of our patient to receive hormone therapy or conventional CT, was an unusual situation out of the context of a clinical trial. The combination of hormone therapy and CDKi is currently the standard first-line therapy for HR+ BC. Pivotal phase III clinical trials with CDKi reported median PFS rates of 24.8 months for letrozole + palbociclib [PALOMA-2 (23)], 20.5 months for letrozole + ribociclib [MONALEESA-2 (24)] and 28.2 months for letrozole + abemaciclib [MONARCH-3 (25)].

Information regarding the effectiveness of CDKi in g*BRCA*m BC is limited. A real-world study published by Collins et al. (26) shows that the outcomes with CDKi may be worse in patients with g*BRCA*m, with a shorter time-to-first subsequent therapy or death and a shorter median OS, suggesting biological differences between g*BRCA*m and g*BRCA* wild-type BC. Frenel et al. (27) demonstrated that patients with HR+/Her2- metastatic BC from PADA-1 trial, who were carriers of *BRCA* and *PALB2* germline mutations, seemed to have a poorer benefit from palbociclib plus hormone therapy than non-mutated patients (mPFS 14 months vs. 26.7 months), presumably because of frequent emergence of *ESR1* resistance mutation in this subgroup.

On the other hand, there is also scarce evidence about the efficacy of PARP inhibitors in the first-line setting of HR+ BC, since both OlympiAD and EMBRACA studies mainly included pretreated patients. Rugo et al. (28) published a subanalysis of outcomes in the prespecified patient subgroups from the EMBRACA study, reporting a median PFS of 9.8 months with talazoparib (95% CI 8.5-13.3) in patients that had not received any previous CT, compared to 8.7 months with physician choice CT (95% CI 5.5-18.0). However, it is presumable that a significant proportion of these patients had triple-negative BC, and among those with HR+ tumors, many had previously received hormone therapy. Well-designed studies are required to properly compare the outcomes of CDKi and PARPi as first-line therapy in advanced g*BRCA*m BC.

Although the prognosis of metastatic BC is worse in cases of endocrine-refractory tumors and visceral involvement (29), the oligometastatic presentation of the disease might have played a role in the favorable outcomes observed in our patient.

### 3.2 PARPi in early-stage BC

PARPi have also shown efficacy in early stage disease. In the OlympiA trial (30), 1 year adjuvant olaparib was compared with placebo in g*BRCA*m carriers with Her2-negative BC and a high risk of recurrence. The olaparib arm was superior, with better invasive disease-free survival (iDFS) (HR 0.58, *p* < 0.0001), distant disease-free survival (dDFS) (HR 0.57, *p* < 0.0001), and OS (HR 0.68, *p* = 0.009). According to these results, not only metastatic patients, but also newly diagnosed patients with localized high-risk disease, should undergo germline testing if a PARP inhibitor may be used for treatment.

The OlympiA eligibility criteria included patients with TNBC and high-burden HR+ BC with residual disease after neoadjuvant CT, as well as patients directly undergoing surgery who had an HR+ tumor with at least four involved axillary nodes, TNBC > 2 cm, or with any axillary involvement. Our patient, who suffered an early relapse of HR+ BC during adjuvant hormone therapy, would not have met the inclusion criteria of the OlympiA study, whose patients with HR+ tumors comprised a particularly high-risk cohort -with a 3-year iDFS of 77% in the placebo arm (15)-. It is inevitable to question whether adjuvant PARPi may have prevented or delayed relapse in our case. Further research is necessary to assess whether a larger population of g*BRCA*m carriers with HR+ BC, with lower-volume tumors or fewer involved axillary nodes than the OlympiA RH+ population, could also benefit from adjuvant PARPi.

Another question relates to the possible role of PARPi in the neoadjuvant setting, which may allow de-escalation or even omission of CT in some g*BRCA*m carriers, especially those with lower-risk BC. Comparable pCR rates were observed with paclitaxel/carboplatin and paclitaxel/olaparib in the GeparOLA trial (31). The use of PARPi alone as neoadjuvant treatment has also been explored, with a 48% pCR rate with talazoparib in g*BRCA*m carriers with TNBC in the NeoTALA trial (32).

Several studies have demonstrated that patients with *BRCA1/2* mutations are more sensitive to cytotoxic drugs that induce DSBs, mainly platinum analogs, because of the deficiency of the HR system. The TNT trial (33) showed that *BRCA*m patients had an increased ORR with carboplatin compared to docetaxel (68% vs. 33%). In both the Geparsixto (34) and CALGB40603 (35) trials, the addition of neoadjuvant carboplatin in TNBC achieved a higher pathologic complete response (pCR). Available data support the efficacy of combining PARPi and platinum in *BRCA*m metastatic BC (36), though phase III trials are warranted to approve its clinical use.

However, there is growing evidence regarding the association between previous platinum exposure and lower response rates to PARPi. In the Olympia trial (30), the improvement in invasive disease-free survival was significantly lower among patients who had previously received platinum-based chemotherapy (HR 0.52) than in platinum-naïve patients (HR 0.77). Desnoyers et al. (37) published a meta-regression analysis of 43 studies, confirming that previous platinum-based treatment was also associated with a lower ORR (*p* = 0.02) in patients with metastatic BC.

### 3.3 The future of PARPi in BC

Several questions remain unanswered regarding the optimal use of PARPi, both in metastatic and (neo)adjuvant scenarios. Further research is required to explore the possible role of PARPi in combination with other systemic therapies, such as CDKi in HR+ tumors and immune checkpoint inhibitors (ICIs) in TNBC. In advanced BC, the combination of PARPi with ICIs -in g*BRCA*m carriers- has shown promising results, for both olaparib plus anti-PDL1 durvalumab [MEDIOLA trial (38)] and niraparib plus anti-PD1 pembrolizumab [TOPACIO/Keynote162 trial (39)]. In the adjuvant setting, up to 12.5% of TNBC patients from the OlympiA study still had distant recurrence even after an accurate treatment with olaparib and intense CT, leaving a wide room for improvement in results, which may be achieved by the addition of ICIs to PARPi. This combination may seem reasonable in TNBC with a large tumor size, nodal involvement, or residual disease after neoadjuvant CT (40), although prospective data are required to validate this hypothesis.

Some preclinical studies have even brought up the concept of ‘chemopreventive’ PARPi, suggesting their potential benefit in healthy g*BRCA*m carriers to reduce the risk of *BRCA*-related cancers, maybe avoiding early prophylactic surgeries (41). Clinical evaluation of the prophylactic effect of PARPi is challenging, because of the limitations in generating prospective evidence, and the difficulty in assessing the risk of contralateral BC in patients treated with PARPi, since most of g*BRCA*m carriers with BC undergo bilateral mastectomy.

The exponential expansion of PARPi from a restricted group of metastatic patients to a much wider population. regarding their use in early stage disease and even their potential prophylactic role in healthy g*BRCA*m carriers, should be accompanied by careful evaluation of their long-term safety. A recent meta-analysis of 31 randomized controlled trials, including 5693 patients treated with PARPi and 3406 in control groups, demonstrated a significant increase in the risk of myelodysplastic syndrome and acute myeloid leukemia (HR 2.63, *p* = 0.026), although the absolute risk remained low (0.73% vs. 0.47%) (42) and PARPi are generally well-tolerated drugs.

In our patient, with no adverse effects, except for mild hematologic toxicity, interruption of talazoparib has not been considered. However, in long-responding patients with worse treatment tolerance and a negative impact on quality of life, one might consider the use of lower-dose schedules, an intermittent exposure to the drug, or even a temporary interruption of PARPi. Prospective studies are needed to explore whether these are feasible strategies, and their impact on clinical outcomes.

To our knowledge, our patient has the longest response to a PARP inhibitor reported to date, as well as the first reported long-term response to talazoparib. Wang et al. (43) have recently published the case of a patient with advanced TNBC and a germline deletion of exon 2 in *BRCA1*, with a complete response to olaparib since September 2017. Exman et al. (44) have reported a partial response to olaparib in a *gBRCA2m* carrier with metastatic BC and leptomeningeal carcinomatosis. Little is known about the biological features of BC or possible predictive factors related to these long-term responses, and further studies are needed to identify this subgroup of patients.

Although somatic *BRCA* mutations (classified as ESCAT IIA) have not the same value as germline alterations (ESCAT I), the potential benefit of PARPi in this subgroup of patients is also a matter of research. In the RUBY trial, rucaparib monotherapy was evaluated in 41 patients with HRR deficiency, including 4 patients with somatic BRCA mutations, reporting 1 partial response and 1 stable disease (45). The TBCRC048 trial evaluated olaparib in 54 patients with metastatic BC and germline mutations in various non-BRCA DNA damage repair genes (cohort 1) and somatic mutations in several genes, including BRCA (cohort 2), with ORR of 33% and 31% respectively (46).

Kuemmel et al. (47) reported a partial response to olaparib in a patient with metastatic HR+ BC harboring a germline sequence variant affecting *PALB2*, supporting the possible benefit of PARPi not only in patients with alterations in *BRCA*, but also in other genes implicated in the HR repair system. Further research is needed to explore whether the benefits of PARPi can be extended to patients harboring germline alterations in other HR-related genes, such as *BARD1* and *RAD51D* mutations or *BRCA* promoter methylation (48).

## 4 Conclusion

Germline mutations in *BRCA1* and *BRCA2* are the most relevant causes of a genetic predisposition to breast cancer. Dysfunction of the homologous recombination repair system makes *BRCA*-deficient cells sensitive to PARP inhibitors, because of the phenomenon of synthetic lethality. In carriers of germline *BRCA* mutations with metastatic BC, olaparib and talazoparib achieved better median PFS than conventional CT, according to the results of the phase III trials OlympiAD and EMBRACA. Olaparib has also shown efficacy as an adjuvant therapy for triple-negative and high-risk HR+ tumors. Neoadjuvant olaparib may be useful to increase the rate of pCR and allow de-escalation or omission of conventional CT in some g*BRCA*m carriers.

Further research is required to answer open questions regarding the use of PARPi, such as their benefit as adjuvant treatment for lower-risk HR+ BC, their possible combination with other systemic therapies, their potential role as prophylactic agents for healthy g*BRCA*m carriers, biological predictive markers of long-term responses, feasibility of de-escalation strategies in long responders, and efficacy in patients with other alterations involving the HR repair system beyond germline *BRCA* mutations.

## Data availability statement

The original contributions presented in the study are included in the article/supplementary material. Further inquiries can be directed to the corresponding author.

## Ethics statement

Written informed consent was obtained from the participant/patient(s) for the publication of this case report.

## Author contributions

VA - conception of the work, writing and review of literature JC, JP, MS, DR – contribution to writing and review of literature CS, MG, AC, EE, EG, EL, MF – treating oncologists and provision of study material NM – treating oncologist, provision of study material and supervision of the work. All authors contributed to the article and approved the submitted version.
